# Earthworms Modulate Impacts of Soil Heterogeneity on Plant Growth at Different Spatial Scales

**DOI:** 10.3389/fpls.2021.735495

**Published:** 2021-12-23

**Authors:** Michael Opoku Adomako, Wei Xue, Sergio Roiloa, Qian Zhang, Dao-Lin Du, Fei-Hai Yu

**Affiliations:** ^1^Institute of Wetland Ecology & Clone Ecology, Zhejiang Provincial Key Laboratory of Plant Evolutionary Ecology and Conservation, Taizhou University, Taizhou, China; ^2^Institute of Environment and Ecology, Academy of Environmental Health and Ecological Security, School of the Environment and Safety Engineering, Jiangsu University, Zhenjiang, China; ^3^BioCost Group, Biology Department, Universidade da Coruña, A Coruña, Spain; ^4^Department of LISA, University of Twente, Enschede, Netherlands

**Keywords:** clonal plant, environmental heterogeneity, foraging response, *Leymus chinensis*, ^15^N-labeled litter, patch scale

## Abstract

Soil heterogeneity (uneven distribution of soil nutrients and/or other properties) is ubiquitous in nature and can greatly affect plant growth. As earthworm activity can influence nutrient redistribution in the soil, we hypothesize that earthworms may alter the effect of soil heterogeneity on plant growth and this effect may depend on the scale of soil heterogeneity. To test these hypotheses, we grew the clonal grass *Leymus chinensis* in three soil treatments (heterogeneous large vs. heterogeneous small patch vs. homogeneous soil treatment) with or without earthworms [i.e., *Eisenia fetida* Savigny (Lumbricidae, epigeic redworm)]. In the heterogeneous treatments, the soil consisted of patches with and without ^15^N-labeled litter (referred to as high- and low-quality patches, respectively), and in the homogeneous treatment, the soil was an even mixture of the two types of soil patches. Biomass of *L. chinensis* was significantly higher in the high- than in the low-quality patches, showing the foraging response; this foraging response occurred at both scales and under both earthworm treatments. Compared to the homogeneous treatment, the heterogeneous large patch treatment increased biomass of *L. chinensis* without earthworms, but decreased it with earthworms. In contrast, biomass of *L. chinensis* in the heterogeneous small patch treatment did not differ from that in the homogeneous treatment, irrespective of earthworms. Belowground biomass was much greater in the heterogeneous small than in the heterogeneous large patch treatment without earthworms, but it did not differ between these two scale treatments with earthworms. In the heterogeneous treatments, soil ^15^N was greater in the high- than in the low-quality patches, but this effect became much weaker with than without earthworms, suggesting that earthworm activity homogenized the soil. We conclude that earthworms can change the impact of soil heterogeneity on plant growth *via* homogenizing the soil, and that this effect of earthworms varies with patch scale. Such scale-dependent interactive effects of soil heterogeneity and earthworms could be a potential mechanism modulating plant community structure and productivity.

## Introduction

In terrestrial ecosystems, strategies for soil nutrient acquisition are important for plants as soil nutrients are commonly spatially heterogeneously distributed (Hutchings and Wijesinghe, 2008; Qian et al., 2014; Liu et al., 2020). Soil nutrient heterogeneity has been shown to have strong impacts on individual plant growth (Birch and Hutchings, 1994; Wijesinghe and Hutchings, 1999; Zhou et al., 2012), population structure (Day et al., 2003a), community productivity (Maestre and Reynolds, 2006; Van Groenigen et al., 2014), intraspecific and interspecific interactions (Zhou et al., 2012; Xue et al., 2018), and species coexistence (Maestre et al., 2005). One underlying mechanism is that some plants show foraging responses by proliferating roots or ramets (asexual individuals of clonal plants) in high-nutrient microsites (Hutchings and de Kroon, 1994; Hodge, 2004). However, earthworm activity may affect soil nutrient distribution (Wurst et al., 2003; Wei et al., 2020), which may alter the impact of soil nutrient heterogeneity on plant growth (García-Palacios et al., 2014; Liu et al., 2020). To date, however, there is little empirical evidence that earthworms can influence the impact of soil nutrient heterogeneity (García-Palacios et al., 2014; Liu et al., 2020). Additionally, it is still unknown whether such an effect of earthworms depends on the scale of nutrient heterogeneity since both earthworm activity and plant responses to soil heterogeneity may be scale-dependent (Wijesinghe and Hutchings, 1999).

The input of litter into soil and its subsequent decomposition and nutrient release are an important source of soil nutrients in ecosystems (Esperschütz et al., 2011; Krishna and Mohan, 2017). Litter is not evenly distributed in ecosystems and an accumulation of litter in specific microsites is common (Loydi et al., 2013; Cai et al., 2019). If litter is decomposed in the places where it is located by soil microbes such as fungi and bacteria, then the patchy distribution of litter will likely result in a patchy distribution of soil nutrients (Bontti et al., 2009; Berg, 2014). However, litter can also be consumed and moved by meso-decomposers such as earthworms, which can reduce soil nutrient heterogeneity associated with the patchy distribution of litter (Hodge et al., 1998; Frouz et al., 2015b).

Earthworms can greatly alter the structure and fertility of soil for plant growth (Mudrák and Frouz, 2018). The movement of earthworms can create channels that increase soil porosity and aeration (Eisenhauer, 2010; Pagenkemper et al., 2015). Earthworms can increase the organic matter content of soil by burying decomposable materials beneath soil for further degradation (Wurst et al., 2003; Wei et al., 2020; Hoeffner et al., 2021). Also, earthworm casting can indirectly increase nitrogen (N) and phosphorus (P) availability by modifying the abundance of soil microbes (Ros et al., 2017; Vos et al., 2019) that are responsible for mineralization of organically bound N and P in the soil (Groffman et al., 2015; Blakemore and Hochkirch, 2017). The movement, consumption of humus and other organic matter, and excrement of earthworms may homogenize patchiness resulting from uneven litter input (Liu and Zou, 2002; Kreuzer et al., 2004). As a result, the presence of earthworms may weaken the effect of spatial heterogeneity of soil on plant growth (Wurst et al., 2003; García-Palacios et al., 2014; Liu et al., 2020).

Patch size and contrast are two inherent components of spatial environmental heterogeneity and can determine its impact on plant growth (Jackson and Caldwell, 1993; Wang et al., 2016). Plant foraging responses to environmental heterogeneity are dependent on both patch scale and patch contrast; plants sensitive to soil heterogeneity at one scale or contrast may not be so at another (Wijesinghe and Hutchings, 1997; Wang et al., 2016). Thus, the effects of environmental heterogeneity on plant growth will vary with patch size and patch contrast (Fransen et al., 1998; Qian et al., 2014; Wang et al., 2016). While several studies have tested the effect of patch size on plant responses to environmental heterogeneity or the effect of earthworms on plant responses to soil heterogeneity (García-Palacios et al., 2014; Liu et al., 2017, 2020), we still lack information on how patch size affects the interactive effects of earthworms and soil heterogeneity created by patchy distribution of litter on plant growth. As earthworm activities are expected to reduce the contrast between patches, we expect that plants will show reduced responses to soil heterogeneity at a certain patch scale in the presence of earthworms as compared to the absence of earthworms.

We grew the rhizomatous grass *Leymus chinensis* in three soil treatments, i.e., two heterogeneous treatments differing in patch size and each consisting of soil patches with or without ^15^N-labeled litter and a homogeneous treatment with an even distribution of the ^15^N-labeled litter. The total amount of the ^15^N-labeled litter in the heterogeneous and homogeneous conditions was exactly the same. We crossed the three soil treatments with the presence or absence of the common litter feeder earthworm *Eisenia fetida*. We addressed the following questions: (1) Does soil heterogeneity created by litter addition affect the growth and foraging response of *L. chinensis*? (2) Does patch scale of such soil heterogeneity matter to plant growth? (3) Do earthworms alter the effect of such soil heterogeneity on the growth and foraging response of *L. chinensis*? (4) Does patch scale affect the impacts of earthworms on the growth and foraging response of *L. chinensis* to soil heterogeneity? We also quantified ^15^N in soil in patches with and without litter addition to indicate the movement of the nutrients due to earthworm activities.

## Materials and Methods

### Study Species

*Leymus chinensis* (Trin.) Tzvelev. is a perennial, rhizomatous, C3 grass, and is widely distributed across the eastern region of the Eurasian steppe (Zhong et al., 2019). It is a dominant species in large areas of this region and sometimes accounts for 80–90% of biomass of the whole community (Wang et al., 2019). It can reproduce both sexually by seeds and asexually by clonal growth *via* rhizomes (Wang et al., 2019). This species has a broad ecological range and high tolerance of drought salinity and low nutrient availability (Bai et al., 2008). Therefore, the species can alleviate losses caused by environmental stress *via* increased production of individual ramets biomass (Zheng et al., 2019). Under conditions of heterogeneous distribution of soil nutrient, *L. chinensis* exhibits strong root foraging capacity for the exploitation of patchily distributed soil nutrient to increase growth, particularly on belowground (Gao et al., 2012; Adomako et al., 2021).

In the autumn of 2018, seeds of *L. chinensis* were collected in grasslands in Inner Mongolia (44.5°N and 115.9°E), China, i.e., one part of the Eurasian steppe. On 7 September 2019, the seeds of *L. chinensis* were sterilized with 5% sodium hypochlorite for 1 min, and rinsed five times with distilled water. The sterilized seeds were then sown in a tray filled with sterilized peat in a greenhouse at Taizhou University, Zhejiang Province, China. Tap water was added daily to promote germination. Seedlings about 6–7 cm tall with 3–5 fully opened leaves were used for the experiment. Earthworms were obtained from a commercial supplier in Zhenjiang, Jiangsu Province, China, and were maintained in organic soil and peat for 3 weeks before the start of the experiment.

### ^15^N-Labeled Litter

On 28 October 2018, 1.496 g of *Lolium perenne* seeds (about 720 seeds; 3,000 seeds m^−2^) was sown in each of eight plastic boxes (60 cm long × 40 cm wide × 23 cm high) filled with an 1:1 (*v*:*v*) mixture of washed river sand and peat. The seeds started to germinate 3 days after sowing and the ^15^N labeling treatment started on 11 November 2018, when seedlings were approximately 7–10 cm in height. We added 725.5 ml commercial water-soluble fertilizer (Peters Professional 20-20-20 General Purpose Fertilizer, Everris, NA, Inc.: 20% total N, 20% available PO_4_, 20% soluble potash, 0.05% Mg, 0.05% Fe, 0.025% Mn, 0.025% Zn, 0.0125% B, 0.0125% Cu, and 0.005% Mo) containing 0.1743 g N and 0.095 g ^15^N in the form of K^15^NO_3_ to each box per week for 10 weeks. In total, we added to each box a total of 2.693 g N, equaling 112 kg N ha^−1^ year^−1^. The atom% of ^15^N was 99.29% in K^15^NO_3_.

The plants were watered regularly until treatment was completed. Five days after the last ^15^N treatment, the aboveground portion (leaves plus vertical stem) of *L. perenne* were harvested and oven-dried at 65°C for 48 h for use as litter. Three samples of the litter material were ground into powder (MM 400, Retsch, Germany) and analyzed for atom% of ^15^N and total N using a DELTA V Isotope Ratio Mass Spectrometer [Thermo Fisher Scientific (Bremen) GmbH, Germany]. The litter samples contained 8.034 ± 1.45 mg/g N and 4.5 ± 0.55 atom% ^15^N (mean ± SE). The rest of the litter was cut into pieces 1 cm long and used for the experiment described below.

### Experimental Design

**Figure 1:**
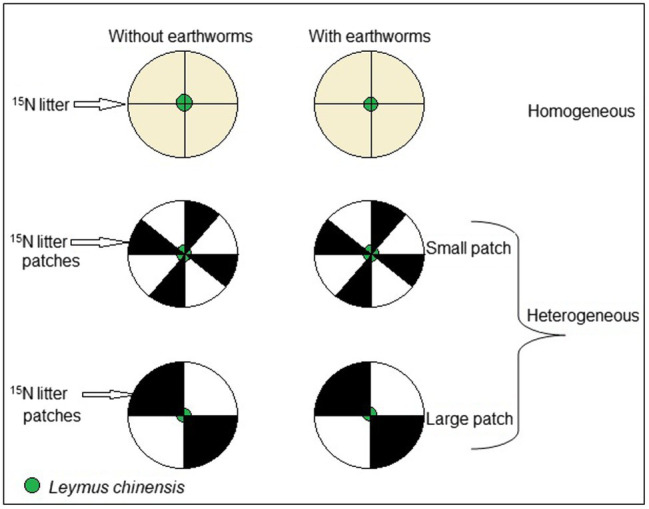
Schematic representation of the experimental design. The experiment consisted of three soil treatments (homogeneous vs. heterogeneous small patch vs. heterogeneous large patch) crossed with two earthworm treatments (with vs. without *Eisenia fetida*). The heterogenous treatments consisted of high-quality patches with ^15^N-labeled litter and low-quality patches without the litter and the homogenous treatments were a even mixture of the high- and the low-quality patches; thus, the nutrients and substrate were exactly the same at the whole pot level for all treatments. Each pot was divided into four large patches (quadrants) in the large patch treatments and eight small patches in the small patch treatments. A mother (initial) ramet of *Leymus chinensis* were grown in the middle of each pot.

The experiment consisted of three soil treatments (homogeneous soil and heterogeneous soil with small or large patches) crossed with two earthworm treatments (with or without), resulting a total of six treatments. Each treatment was replicated six times, making 36 pots in total. We filled each pot from the bottom to the top with quartz sand, mineral soil and organic soil to mimic the normal profile of grassland soils as described below. In the large patch treatment, each pot (32 cm × 24 cm, diameter × height) was partitioned into four equal quadrants using a divider. However, the divider was removed after the pot is filled and the patches created to ensure free root growth and earthworm movement across patches. Two opposite quadrants of the pot were each filled, from the bottom to the top, as follows: (1) the bottom of the pot was filled with 150 ml quartz sand [particle size of 0.5–1 mm; Sinoteng Silica Materials Technology (Jiangsu) Co. Ltd., Xuzhou, China]. (2) We added 250 ml mineral soil [1:1 (v:v) of nutrient-poor local soil (total *N* = 0.779 ± 0.271 g kg^−1^; total *p* = 0.067 ± 0.044 g kg^−1^; pH = 7.1 ± 0.26; clay = 11.7 ± 0.1%; silt = 8.5 ± 0.12%; sand = 79.8 ± 0.08%; *n* = 3) and washed river sand] on top of the quartz sand to constitute the subsoil. (3) To create the topsoil, we added 500 ml organic soil [1:1 (*v:v*) of peat (Recipe 413, a lightly decomposed oligotrophic peat with a pH of 5.5–6.5 from Lithuania, Klasmann-Deilmann Produktionsgesellschaft Nord GmbH & Co., Saterland, Germany) and grassland topsoil (total *N* = 5.28 ± 1.52 g kg^−1^; total *p* = 0.26 ± 0.01 g kg^−1^; pH = 7.2 ± 0.07; clay = 20.4 ± 0.64%; silt = 9.6 ± 0.23%; sand = 70 ± 0.35%; *n* = 3)] collected between 0 and 15 cm layer from Inner Mongolia (44.5°N, 115.9°E). In the patches with added litter, 3.5 g of ^15^N labeled litter was mixed into the top 5 cm layer of the organic soil (Figure 1). However, the other two quadrants of the pot were filled in the same way, except that no ^15^N labeled litter was added. It is worth mentioning that peat was added to the organic soil as food source for earthworms that may be located in the treatment without the added ^15^N labeled litter. We arranged the soil in the pot, in this manner, to mimic the structure of a typical grassland soil.

In the small patch treatment, each pot was partitioned into eight equal patches and four out of these eight patches were each filled similarly to the large patches, from the bottom to the top. At the bottom level, we added 75 ml of the quartz sand, followed by 125 ml of the mineral soil as the subsoil, and 250 ml of the organic soil as the topsoil. Also, in the patches with added litter, 1.75 g of ^15^N labeled litter was added to the top 5 cm layer of the organic soil; however, the other four patches of the pot were filled in the same way, except that no ^15^N labeled litter was added (Figure 1).

In the homogeneous treatment, each pot was filled in the same way as the heterogeneous treatments, except that a total of 7.0 g of ^15^N labeled litter was evenly mixed into the top 5 cm layer of the organic soil so that the nutrients was evenly distributed. In this way, total N in ^15^N labeled litter (*ca*. 56 mg N) per pot was the same in all three soil treatments. Prior to removing the divider after filling a pot, we marked the end of opposite quadrants on top of the pot to facilitate patch harvesting of both the above- and belowground biomass.

On 1 October 2019, one seedling of *L. chinensis* was planted in the middle of each pot (Figure 1). In the treatments with earthworms, 12 adult individuals of *E. fetida* were released into each pot. This provided a density of 100–120 individuals/m^2^, within the range of earthworm densities in most arable soils (Smith et al., 2008). Fresh biomass (including gut content) of the earthworms added at the start of the experiment was 0.61 ± 0.01 g (mean ± SE, *n* = 18); their average length was 6.5 cm.

We arranged the pots randomly in a greenhouse at the Jiaojiang campus of Taizhou University, Taizhou, Zhejiang Province, China. During the experiment, we randomly re-arranged the pots every 3 weeks to avoid the potential effects of micro-environmental heterogeneity. The mean temperature in the greenhouse was 26.1°C and the humidity was maintained at about 85%. The plants were watered regularly. The experiment lasted 123 days, starting on 1 October 2019 and ending on 1 February 2020.

### Harvesting and Measurement

On 1 February 2020, the aboveground portion (leaf blades plus aboveground vertical stems) and belowground portion (roots) of the mother (initial) plant were harvested separately. Likewise, the aboveground portion (leaf blades plus aboveground vertical stems) and belowground portion (roots plus rhizomes) of offspring ramets produced in quadrants with and without added litter in the heterogeneous treatments and imagined quadrants with and without added litter in the homogeneous treatments were also harvested separately, and oven-dried at 70°C for 72 h to measure dry mass. In the two heterogeneous treatments, the quadrants with ^15^N-labeled litter are thereafter referred to as high-quality patches, whereas those without ^15^N -labeled litter are referred to as low-quality patches. In the homogeneous treatments, the imagined quadrants are named similarly.

The earthworms that were still alive were counted, frozen, dried (by subliming the ice through heating), and weighed to obtain the freeze-dry weight for each treatment. The earthworm weight change (freeze-dry weight at harvest *minus* estimated initial freeze-dry weight) was recorded (García-Palacios et al., 2014). This freeze-dry method reduces the possibility of losing parts of the earthworm’s soft body. To examine the transfer of ^15^N-labeled litter across patches due to earthworm movement, soil samples were taken from one randomly selected high-quality patch and its adjacent low-quality patch in each pot. After air-drying, the soil samples were ground into powder and analyzed for atom% of ^15^N using a DELTA V Isotope Ratio Mass Spectrometer [Thermo Fisher Scientific (Bremen) GmbH, Germany].

### Statistical Analysis

We calculated biomass (total, aboveground, and belowground) of *L. chinensis* in a pot by summing biomass (total, aboveground, and belowground) of the mother plant and of the offspring ramets produced in the pot. At the patch level, we obtained total, aboveground, and belowground biomass of offspring ramets in the high- and low-quality patches separately. We also computed the percentage of earthworms recovered and their weight change.

We used two-way ANOVAs to test the effects of soil treatments (homogeneous treatment vs. heterogeneous small patch treatment vs. heterogeneous large patch treatment), earthworms (with vs. without) and their interaction on total, aboveground, and belowground biomass of *L. chinensis* in the whole pot. *Post hoc* Turkey tests were used to compare means among the three soil treatments within each earthworm treatment. At the patch level, we used three-way ANOVAs to examine the effects of soil treatments, earthworms, patch quality (high vs. low quality) and their interactions on total, aboveground and belowground biomass of the offspring ramets of *L. chinensis* and on ^15^N in soil. In the model, patch quality was treated as a repeated variable as the data in the high- and low-quality patches in the same pots were not independent (Dong et al., 2015; Xue et al., 2018). Planned contrasts were used to compare means between the high- and low-quality patches within each soil and earthworm treatment (Sokal and Rohlf, 1995).

Before analysis, all data were checked for homogeneity of variance by Levene’s test and normality by K-S test. Data on biomass and ^15^N at the patch level were logarithmically transformed before ANOVAs. As data did not meet the requirements of ANOVA even after transformation, we used Kruskal–Wallis rank tests to examine the differences in the percentage of earthworms recovered and their weight change between the homogenous, the heterogeneous large patch, and the heterogeneous small patch treatment. All data were analyzed using R v3.6.1 (R Core Team, 2019).

## Results

### Whole Plant Growth at the Pot Level

**Figure 2:**
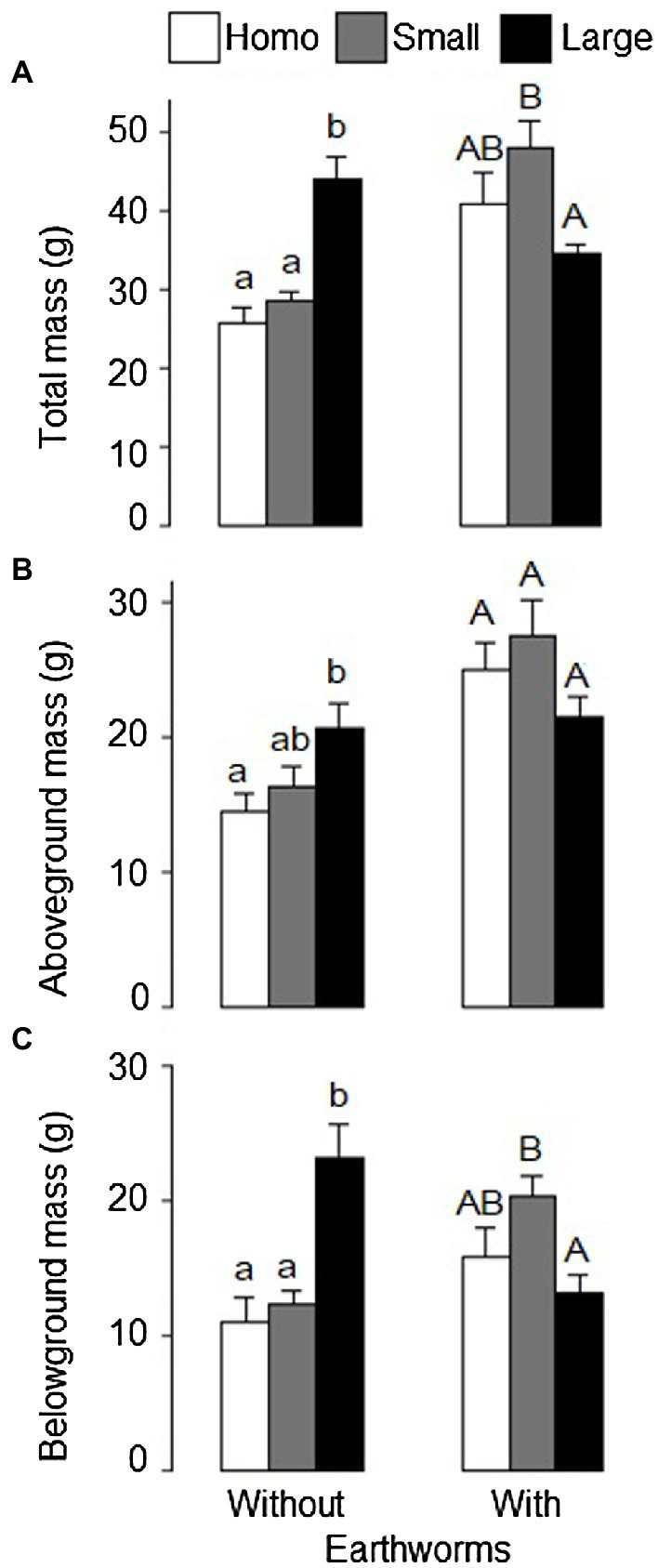
**(A)** Total, **(B)** aboveground, and **(C)** belowground mass of *Leymus chinensis* under the homogeneous, heterogeneous small patch and heterogeneous large patch treatments with or without earthworms. Mean ± SE (*n* = 6) are given. Different letters indicate significant difference within each earthworm treatment (by *Tukey* test).

Effects of soil treatment on total, aboveground and belowground biomass of *L. chinensis* depended significantly on the presence of earthworms (Table 1: significant effects of soil treatment × earthworms). In the absence of earthworms, total biomass and belowground biomass were significantly greater in the heterogeneous, large patch treatment than in the heterogeneous, small patch treatment and the homogeneous treatment (Figures 2A,C).

**Table 1:** Results of ANOVAs for effects of soil treatment (homogeneous vs. heterogeneous small vs. heterogeneous large scale), earthworm (with vs. without) and their interactions on total, aboveground and belowground mass of *Leymus chinensis* at the whole pot level.

Effect	DF	Total mass	Aboveground mass	Belowground mass
Soil treatment (S)	2, 30	*2.7^#^*	0.6^ns^	**3.5** ^*^
Earthworm (E)	1, 30	**13.8** ^***^	**23.4** ^***^	0.3
S × E	2, 30	**15.6** ^***^	**4.7** ^*^	**14.2** ^***^

F values and significance levels (^***^p < 0.001, ^**^p < 0.01, ^*^p < 0.05, ^#^p < 0.1 and ^ns^p ≥ 0.1) are given. Values are in bold when p < 0.05 and in italics when p < 0.1.

In the presence of earthworms, however, total biomass and belowground biomass were significantly greater in the small patch treatment than in the large patch treatment and these two variables in the homogeneous treatment did not differ from those in either the large or the small patch treatment (Figures 2A,C). Quantitatively, aboveground biomass showed the similar pattern as total and belowground biomass (Figure 2B).

### Offspring Growth at the Patch Level

**Figure 3:**
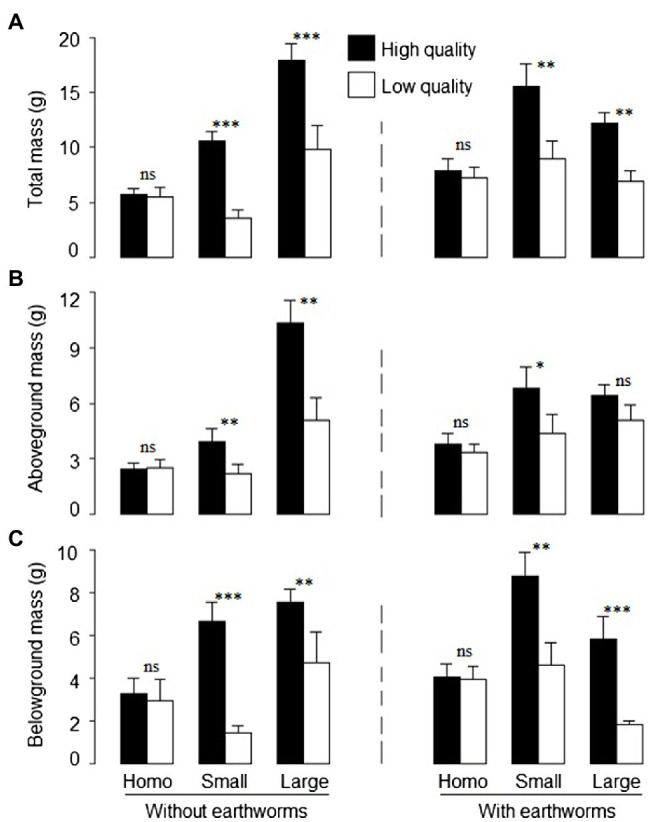
**(A)** Total, **(B)** aboveground, and **(C)** belowground mass of offspring ramets of *Leymus chinensis* in the high- and low-quality patches in the heterogeneous small patch and large patch treatments and in the imagined high- and low-quality patches in the homogeneous treatments with or without earthworms. Mean ± SE (*n* = 6) are given. Symbols above pairs of bars shows significance levels (^***^p < 0.001, ^**^p < 0.01, ^*^p < 0.05 and ^ns^*p* > 0.05; by linear contrast).

Patch quality significantly affected total, aboveground and belowground biomass of *L. chinensis*, and such effects depended on soil treatment (Table 2: significant or marginal significant effects of soil treatment × patch quality), but was independent of the presence of earthworms (Table 2: no significant effect of earthworm × patch quality or soil treatment × earthworm × patch quality). Irrespective of the earthworm presence, total biomass and belowground biomass did not differ between the imagined high- and low-quality patches in the homogeneous treatment, but were much greater in the high- than in the low-quality patches in both the large and the small patch treatment (Figures 3A,C). Quantitatively, aboveground biomass showed the similar pattern as total and belowground biomass (Figure 3B).

**Table 2:** Results of ANOVAs for effects of soil treatment (homogeneous vs. heterogeneous small vs. heterogeneous large scale), earthworm (with vs. without) and patch quality (with vs. without added litter) and their interactions on total, aboveground and belowground mass of offspring ramets of *Leymus chinensis* and ^15^N atomic percentage and N concentration in soil at the patch level.

Effect	DF	Total mass	Aboveground mass	Belowground mass	Soil ^15^N
*Between-subject*					
Soil treatment (S)	2, 30	**6.6** ^***^	**7.7** ^**^	1.3^ns^	1.0^ns^
Earthworm (E)	1, 30	*3.3* ^#^	2.6^ns^	2.0^ns^	**6.2** ^*^
S × E	2, 30	**6.1** ^**^	2.1^ns^	**6.8** ^**^	0.2^ns^
*Within-subject*					
Patch quality (Q)	1, 30	**49.4** ^***^	**15.5** ^***^	**48.4** ^***^	**46.9** ^***^
S × Q	2, 30	**10.3** ^***^	*3.0* ^#^	**9.2** ^***^	**12.5** ^***^
E × Q	1, 30	2.2^ns^	0.9^ns^	1.1^ns^	**5.8** ^*^
S × E × Q	2, 30	1.3^ns^	0.7^ns^	*2.6* ^#^	*2.7* ^#^

Data were logarithmically transformed before analysis. F values and significance levels (^***^p < 0.001, ^**^p < 0.01, ^*^p < 0.05, ^#^p < 0.1 and ^ns^p ≥ 0.1) are given. Values are in bold when p < 0.05 and in italics when p < 0.1.

### Soil ^15^N at the Patch Level

**Figure 4:**
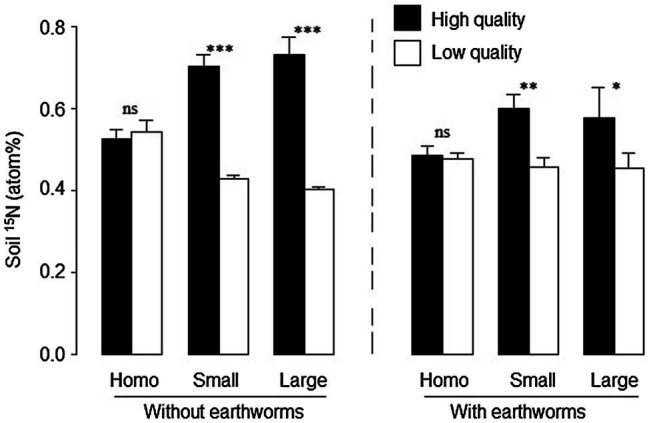
Soil ^15^N in the high- and low-quality patches in the heterogeneous small patch and large patch treatments and in the imagined high- and low-quality patches in the homogeneous treatments with and without earthworms. Mean ± SE (*n* = 6) are given. Symbols above pairs of bars shows levels of differences between the high- and low-quality patches (^***^p < 0.001, ^**^p < 0.01, ^*^p < 0.05 and ^ns^*p* > 0.05; by linear contrast).

Averaged across the other treatments, soil ^15^N was significantly lower with than without earthworms (significant effect of earthworm in Table 2 and Figure 4). Patch quality significantly affected soil ^15^N atomic percentage, but such an effect depended on both soil treatment and earthworms (Table 2: significant effects of soil treatment × patch quality and earthworm × patch quality). In the homogeneous treatments, soil ^15^N did not differ between the imagined high- and low-quality patches, no matter whether earthworms were present or not (Figure 4). In the heterogeneous treatments, soil ^15^N was much greater in the high- than in the low-quality patches, and such an effect was much stronger without than with earthworms (Figure 4).

### Earthworm Number and Biomass

**Figure 5:**
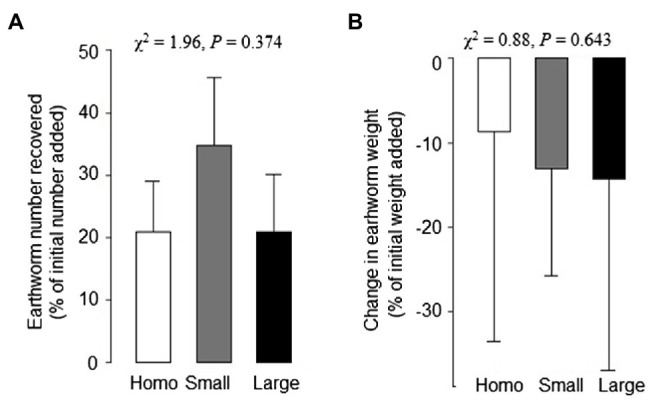
Changes in number **(A)** and freeze-dry weight **(B)** of earthworms in the homogeneous, heterogeneous large patch and heterogeneous small patch treatments. Mean ± SE (*n* = 6) and *χ*^2^ and values of *p* of Kruskal–Wallis rank test are given.

At harvest, the number of earthworms recovered and the earthworm biomass were reduced by about 75 and 15%, respectively, compared to values at the start of the experiment (Figures 5A,B). However, soil treatments had no significant impact on the changes in the number of earthworms recovered or biomass of the earthworms (Figures 5A,B).

## Discussion

Soil heterogeneity, often observed at different spatial scales, is a common characteristic of natural habitats (Lechowicz and Bell, 1991). Vegetative spreading allows clonal plants to expand across patchy areas, and a well-known effect of this soil heterogeneity is a foraging response that can increase plant growth (Birch and Hutchings, 1994; Hutchings and Wijesinghe, 2008; Zhou et al., 2012). Our study demonstrated that the effect of heterogeneous distribution of plant litter on the performance of the clonal grass *L. chinensis* was significantly affected by the presence of earthworms, and such an effect of earthworms also depended on the spatial scale of heterogeneity. As far as we know, this is the first experimental evidence showing a scale-dependent effect of earthworms on plant responses to soil heterogeneity.

### Interactive Effects of Earthworms and Litter Heterogeneity on Whole Plant Growth at the Pot Level

Compared to homogeneous distribution of litter, heterogeneous distribution in large patches greatly increased biomass of *L. chinensis* in the absence of earthworms, but decreased it in the presence of earthworms (Figure 2). In contrast, heterogeneous distribution of litter in small patches did not increase plant growth relative to homogeneous distribution, regardless of the presence of earthworms (Figure 2). These results suggest that the effect of heterogeneous distribution of litter on plant growth strongly depended on the presence or absence of earthworms and the scale of heterogeneity, and that the effect of earthworms on the responses of plants to litter heterogeneity varied with the spatial scale of heterogeneity. Thus, the results reported in our study provide empirical evidence of the scale-dependent interactive effect of earthworms and soil heterogeneity on plant growth.

Previous studies have shown that plants with a high ability of root foraging can exploit a higher amount of nutrients from nutrient-rich patches and such a plastic ability has been shown to increase with increasing patch size (Wijesinghe and Hutchings, 1997; Dong et al., 2014; Qian et al., 2014; Wang et al., 2016). The reason is that large patches have higher nutrient contrast compared to small patches, which will likely compel the plant to introduce more roots and ramets into the large patches for greater nutrient uptake. Therefore, the positive effects of patch contrast will equally benefit plants in the large patches than those in the small patches. In the present study, we found evidence of this positive effect of soil heterogeneity and root foraging. In the absence of earthworms, biomass of *L. chinensis* was higher in the heterogeneous, large patch treatment than in the homogeneous treatment. However, the introduction of earthworms altered these positive effects of soil heterogeneity, and we observed a reduction of plant growth in the heterogeneous, large patch treatment compared to the homogeneous treatment in the presence of earthworms.

The presence of earthworms increased plant biomass when litter was homogeneously distributed, but decreased it when litter was heterogeneously distributed in large patches (Figure 2). This is one of the few studies where earthworms have reduced plant biomass in heterogeneous conditions compared with homogeneous distribution (see Poor et al., 2005; García-Palacios et al., 2014). A plausible explanation for the earthworm reduction of plant biomass at large-scale heterogeneous soils is that the presence of earthworms reduces the contrast between patches. Consequently, the benefit gained from heterogeneous distribution of, e.g., nutrients disappears, and plants show a reduction of total biomass. Earthworm activity homogenizes nutrient-rich patches (similar to the large patches in this study) by moving available nutrients from high-nutrient zones to nutrient-poor zones (Rossi et al., 1997; Knowles et al., 2016). For this reason, earthworm activity tends to have a strong positive impact on plant growth when nutrient levels are low rather than when they were high (Knowles et al., 2016; Frouz, 2018). In addition to the reduction in soil nutrient heterogeneity, earthworm activity can also alter soil structure and increase nutrient availability, which consequently increases plant growth. One explanation for our results is that the costs incurred by *L. chinensis* due to the reduction of soil heterogeneity by earthworm activity was higher than the benefits gained by the presence of the earthworms. As a result, plants experienced a biomass reduction when earthworms were present at the large-scale heterogeneous soil.

Contrary to the pattern observed in the large-scale heterogeneous soil, the presence of earthworms significantly increased total biomass (by 67%) of *L. chinensis* growing in the small-scale heterogeneous soil. A plausible explanation for this result is that, at this small-scale heterogeneity, the benefit gained from the earthworm activity (i.e., soil fertility increase) is higher than the cost incurred by the presence of the earthworms (i.e., reduction of soil heterogeneity). Because the benefit of soil heterogeneity for plant growth has been shown to be greater at larger than at smaller patch size (Wijesinghe and Hutchings, 1997; Dong et al., 2014; Wang et al., 2016), we can expect that at small scale, the benefit of soil heterogeneity could be lower than the benefit derived from the presence of the earthworms, even when the presence of earthworms can homogenize the soil.

Our results suggest that effects of earthworms on soil nutrient heterogeneity due to heterogeneous litter input could be a potential mechanism modulating plant growth and consequently plant community structure. Our findings also suggest that soil nutrient heterogeneity may reliably increase plant growth only when the populations of organisms with strong effects on soil nutrients and conditions (i.e., soil fauna such as earthworms, termites and millipedes) are limited (Fujimaki et al., 2010; Cheik et al., 2019).

### Interactive Effects of Earthworms and Litter Heterogeneity on Offspring Growth at the Patch Level

The growth of *L. chinensis* was significantly greater in the high-quality patches with litter than in the low-quality patches without litter, and this pattern was consistent at both scales of soil heterogeneity and with and without earthworms (Figure 3). The decomposition of nutrient-rich litter can greatly increase nutrient input into the soil (Ge et al., 2013) and thus the soil patches with litter should have a higher nutrient availability than those without litter. It is well known that plants, especially clonal ones, can show stronger growth in favorable patches than in unfavorable patches when they grow in heterogeneous environments (Cahill et al., 2010; Giannopoulos et al., 2010; Cahill and McNickle, 2011). As a result, significantly more biomass and ramets are often distributed in favorable patches than in un-favorable ones. Such a foraging response can, in turn, greatly increase the resource uptake efficiency of the ramets located in the high-nutrient patches and further increase resource harvesting and thus the whole plant growth (Kleijn and van Groenendael, 1999; Day et al., 2003b; Roiloa and Retuerto, 2006b). Similarly, in the present study the greater biomass of *L. chinensis* in the patches with than without litter was likely due to the foraging response of this clonal plant associated with the heterogeneous distribution of soil nutrients mediated through litter decomposition.

While the presence of earthworms did not affect the foraging response in terms of total and aboveground mass of *L. chinensis*, it significantly affected the strength of the foraging response in terms of belowground biomass (Table 2; Figure 3C). We found that the foraging response of belowground biomass was much stronger in the small than in the large patch treatment in the absence of earthworms, but was similar in the small and the large patch treatment in the presence of earthworms (Figure 3C). Interestingly, without earthworms, the stronger effect of soil quality detected in small patches was due, not to differences between belowground biomass in high-quality areas, but to differences detected in low-quality areas (Figure 3C). Thus, while belowground biomass produced in the high-quality patches was similar at small and large heterogeneity, belowground mass in the low-quality patches was much greater at large than at small scale. A potential explanation for this result could be associated to the benefit gained by clonal plants from large-scale heterogeneity (Wijesinghe and Hutchings, 1997; Dong et al., 2014; Wang et al., 2016). Thus, resource sharing between interconnected offspring within the clonal system would result in a benefit for individuals located at low-quality patches (Hartnett and Bazzaz, 1983; Slade and Hutchings, 1987; Yu et al., 2004; Roiloa and Retuerto, 2006a). This benefit was especially evident at large-scale heterogeneity, resulting in smaller difference between high- and low-quality patches at larger scales than at smaller scales. On the other hand, the absence of differences between small and large scales in the presence of earthworms could be explained again by the homogenization effect of soil fauna activity. Thus, the reduction of soil heterogeneity caused by earthworms also reduced the benefit gained by the clone, including belowground biomass produced in the low-quality patches. These results suggest that the foraging responses of plants in environments with heterogeneous distribution of litter and thus nutrients and their subsequent growth could be regulated by the presence of earthworms, as their activity largely determines the stability of nutrient-rich patches (Snyder et al., 2009; Frouz, 2018). The results also indicate a scale-dependent effect of earthworms on the foraging responses of plants in heterogeneous environments.

### Earthworm Performance and Effects of Earthworms on Soil Nutrient Distribution

Because ^15^N-labeled litter was not placed in the low-quality patches at the start of the experiment, the amount of ^15^N found in the low-quality patches at harvest could be used to infer the transfer of litter or nutrients due to earthworm activity, as in other studies (Kreuzer et al., 2004; Frouz et al., 2015a; Xiao et al., 2018). The observed patterns of ^15^N in soils suggest that earthworms can move nitrogen from the high- to the low-quality patches and that earthworm activity can reduce soil nutrient heterogeneity (García-Palacios et al., 2014; Liu et al., 2020).

The transfer of ^15^N from the high-quality to the low-quality patches also provides evidence that earthworms could circulate the organic material of litter or its decomposing parts as they moved within the soil matrix (Liu and Zou, 2002; Friberg et al., 2008; Eisenhauer, 2010). We found a great reduction of the earthworm number at the end of the experiment. In this sense, we recognize that the experimental approach could bring certain limitation for earthworm performance, and therefore extrapolation to field conditions has to be done with caution. Thus, the physical mixing of litter in pots may differ from the mixing of litter by soil fauna (Kreuzer et al., 2004; Liu et al., 2019), and earthworm movement limitations by pots can potentially reduce the performance of earthworms.

## Conclusions

This study reports novel evidence about the scale-dependent interactive effect of earthworm activity and soil heterogeneity on the performance of *L. chinensis*. We conclude that soil homogenization due to the *E. fetida* Savigny activity had greater effects at larger scales of heterogeneity, where the benefit of heterogeneity is expected to be higher, than at smaller scales of heterogeneity. Our findings suggest that earthworms can have important ecological consequences for plant performance in heterogeneous environments, being beneficial or detrimental depending on patch scale. To have a more accurate picture of the interaction between earthworms and soil heterogeneity in a real field situation, field studies that include more patch scales with different functional groups of plants and earthworm species must be considered, as their respective responses may differ accordingly.

## Data Availability Statement

The raw data supporting the conclusions of this article will be made available by the authors, without undue reservation.

## Author Contributions

F-HY, QZ, and D-LD contributed to conception and design of the study. MOA performed the experiments, collected the data, and drafted the manuscript. MOA and WX analyzed the data. F-HY and SR revised the manuscript. All authors contributed to the article and approved the submitted version.

## Funding

This study was supported by the National Natural Science Foundation of China (31800341) and the Ten-Thousand-Talent Program of Zhejiang Province (2018R52016).

## Conflict of Interest

The authors declare that the research was conducted in the absence of any commercial or financial relationships that could be construed as a potential conflict of interest.

## Publisher’s Note

All claims expressed in this article are solely those of the authors and do not necessarily represent those of their affiliated organizations, or those of the publisher, the editors and the reviewers. Any product that may be evaluated in this article, or claim that may be made by its manufacturer, is not guaranteed or endorsed by the publisher.
